# A Comparison of Bioelectric and Biomechanical EMG Normalization Techniques in Healthy Older and Young Adults during Walking Gait

**DOI:** 10.3390/jfmk9020090

**Published:** 2024-05-22

**Authors:** Drew Commandeur, Marc Klimstra, Ryan Brodie, Sandra Hundza

**Affiliations:** 1 Motion and Mobility Laboratory, University of Victoria, Victoria, BC V8P 5C2, Canada; 2 School of Exercise Science, Physical and Health Education, University of Victoria, Victoria, BC V8W 3P2, Canada; 3 Canadian Sport Institute Pacific, Victoria, BC V9E 2C5, Canada; rbrodie@csipacific.ca; 4 International Collaboration on Repair Discoveries (ICORD), Vancouver, BC V5Z 1M9, Canada

**Keywords:** EMG, EMG normalization, biomechanical normalization, bioelectric normalization, ageing, gait

## Abstract

This study compares biomechanical and bioelectric electromyography (EMG) normalization techniques across disparate age cohorts during walking to assess the impact of normalization methods on the functional interpretation of EMG data. The biomechanical method involved scaling EMG to a target absolute torque (EMG_TS_) from a joint-specific task and the chosen bioelectric methods were peak and mean normalization taken from the EMG signal during gait, referred to as dynamic mean and dynamic peak normalization (EMG_Mean_ and EMG_Peak_). The effects of normalization on EMG amplitude, activation pattern, and inter-subject variability were compared between disparate cohorts, including OLD (76.6 yrs N = 12) and YOUNG (26.6 yrs N = 12), in five lower-limb muscles. EMG_Peak_ normalization resulted in differences between YOUNG and OLD cohorts in Biceps Femoris (BF) and Medial Gastrocnemius (MG) that were not observed with EMG_Mean_ or EMG_TS_ normalization. EMG_Peak_ and EMG_Mean_ normalization also demonstrated interactions between age and the phase of gait in BF that were not seen with EMG_TS_. Correlations showed that activation patterns across the gait cycle were similar between all methods for both age groups and the coefficient of variation comparisons found that EMG_TS_ produced the greatest inter-subject variability. We have shown that the normalization technique can influence the interpretation of findings when comparing disparate populations, highlighting the need to carefully interpret functional differences in EMG between disparate cohorts.

## 1. Introduction

The amplitude of raw electromyography (EMG) can be greatly affected by conditions during collection including subcutaneous tissue thickness [1], electrode placement [2], joint angle and muscle movement [3], as well as cross talk from nearby muscles [4]. Therefore, it is not valid to make comparisons of raw EMG amplitude between subjects, muscles, tasks, or after changing the placement of electrodes. To enable comparisons of EMG between individuals, groups and conditions over time during walking gait, EMG is typically normalized using either bioelectric or biomechanical techniques [5]. Bioelectric methods normalize EMG to a level recorded during a task such as walking or an isolated normalization task. Commonly employed bioelectric techniques include maximum voluntary isometric contraction (MVIC), dynamic EMG peak (EMG_Peak_) or dynamic EMG mean (EMG_Mean_), among others [6]. For a detailed comparison of bioelectric methods, see [5]. EMG_Peak_ and EMG_Mean_ are the maximum and mean EMG amplitude achieved during a task, respectively. MVIC is the average maximum EMG amplitude achieved during an isometric contraction using an independent isometric task. One issue of using MVICs to normalize EMG is the lack of consistency between trials, as demonstrated by Yang and Winter (1984) [7], who found that within-day and between-day submaximal contractions are more reliable compared to MVIC. Further, for older individuals, there can be a hesitancy to perform a true MVIC due to discomfort or pain associated with this task [8], particularly if the patients have an age-related injury or pathology [9,10] such as arthritis [11]. Submaximal contractions for normalization of EMG_Peak_ and EMG_Mean_ are less physically strenuous and therefore address the issues of consistency and discomfort associated with the MVIC technique [7,12].

A limitation of bioelectric normalization techniques is that they could result in a misrepresentation of the absolute amplitude of force generated by the muscle and impede apposite comparison between functionally disparate groups [6]. For example, in clinical populations, during a task such as walking, the force generated by a muscle could be much lower than the controls, but this would not be adequately captured using MVIC, EMG_Peak_ and EMG_Mean_ normalization [13].

Bioelectric normalization methods are used to examine the relative differences between individuals or groups, but often an absolute comparison may provide more relevant information concerning force production during a task. Therefore, the use of a biomechanical EMG normalization technique based on torque-scaled values of EMG could enable functionally relevant comparisons between disparate populations [5,14]. This type of normalization technique has been previously employed [7,15,16]. For example, Ng et al. (2002) [15] assessed the difference in the EMG activity of trunk muscles between individuals with and without back pain using submaximal contractions to specific loads. To date, bioelectric and biomechanical methods of normalization have not been rigorously compared to determine the effect of the normalization technique on the capacity to detect differences between disparate cohorts during walking gait. While Yang and Winter (1984) [7] did compare bioelectric and biomechanical methods of normalization, the biomechanical normalization method used a subject relative load (50% MVIC), and thus this method did not incorporate EMG activation associated with an externally determined target absolute load. Therefore, there is a need to compare bioelectrical EMG normalization techniques to a biomechanical EMG normalization method that uses a common target absolute load to detect differences in EMG amplitude and activation pattern between disparate cohorts during walking gait.

This study compared EMG between OLD and YOUNG subjects using an EMG torque-scaled (EMG_TS_) normalization technique and two popular methods of bioelectric EMG normalization, EMG_Mean_ and EMG_Peak_. These methods were compared with respect to their ability to detect differences in EMG amplitude, activation pattern and inter-subject variability during walking gait. Having a better understanding of the limitations and benefits of each normalization technique facilitates a more informed choice that may impact the interpretation of findings. As the current literature provides little evidence to characterize the relationship of the amplitude and activation pattern differences between bioelectric and biomechanical normalization methods, we sought to determine whether the chosen normalization method impacts the functional interpretation of the data. We also expected that the inter-individual variability of EMG would be preserved with biomechanical normalization and reduced with bioelectrical methods.

## 2. Materials and Methods

### 2.1. Participants

Twelve healthy YOUNG (six males, six females, aged 26.6 ± 5.5 yrs) and twelve healthy OLD (eight males, four females, aged 76.6 ± 5.0 yrs) adults were convenience sampled and participated in the study with informed written consent. Subjects were screened using the Canadian PAR-Q+ questionnaire and medical clearance was requested for participants who answered Yes to any question. Inclusion criteria for the study required participants to be able to walk unassisted, have a mini-mental state exam score of 24 or greater, and older participants were aged 65 years or older. Participants were excluded if they were unable to walk at least 200 m or had a musculoskeletal or neurological disorder. The experimental protocol was approved by the University of Victoria Human Research Ethics Committee (12-272).

### 2.2. Protocol

#### 2.2.1. Walking Trials

Subjects walked on a treadmill at a self-selected walking pace (OLD—3.57 +/− 0.58 km/h; YOUNG—4.14 +/− 0.74 km/h) while EMG was recorded from the right lower limb throughout the gait cycle. 

#### 2.2.2. Submaximal Isometric Contraction Trials and Load–EMG Relationship Determination

Subjects were seated in a chair with a padded cuff attached to their right lower leg for the knee flexion/extension task or the foot for ankle plantarflexion/dorsiflexion task. To determine the torque–EMG relationship for the muscles studied, four submaximal isometric contractions were performed with the force of each contraction measured via a load cell (model LC101-50, Omega Sensing Solutions ULC, St Eustache, QC, Canada). For knee flexion and extension, the knee was positioned at an angle of 120° with the cuff 20 cm distal to the knee joint centre; participants produced and maintained torque moments of 0.91 Nm (knee flexion) and 1.36 Nm (knee extension) corresponding to a load of 10 lbs and 15 lbs, respectively. For plantarflexion and dorsiflexion, the subject was standing and facing a padded chair, the knee was placed at 90 degrees on the seat with the ankle held at 90° and the cuff positioned on the foot 10 cm distal to the centre of medial malleolus; subjects produced and maintained torque moments of 0.682 Nm (plantarflexion) and 0.273 Nm (dorsiflexion) corresponding to loads of 15 lbs and 6 lbs, respectively. Knee and ankle joint angles were positioned and maintained by the researcher using a manual goniometer. Participants were instructed to gradually increase the force of contraction to the target force (±0.05 kg) and maintain for 5 s. A digital oscilloscope was used to provide visual feedback to the participant. Familiarization trials were performed until the participant could reliably maintain the target force.

### 2.3. EMG Collection and Conditioning

Electromyographic (EMG) activity was collected using Ag-AgCl bi-polar disposable electrodes (model T3425, Thought Technology, Montreal, QC, Canada) spaced 2 cm from centre-to-centre. EMG electrode sites were prepared by removing body hair and thoroughly cleaning the area with alcohol swabs. EMG was recorded from tibialis anterior (TA), soleus (SOL), medial gastrocnemius (MG), vastus lateralis (VL), and biceps femoris (BF) on the right side. Common grounds were placed over the patellar surface. EMG for both the torque scaled and walking trials were pre-amplified at a gain of 5000 and filtered at 10–300 Hz by Grass Technologies P511 (model P511 Grass Instruments, AstroNova, Brossard, QC, Canada). Recorded EMG was full-wave rectified and then filtered using a 40 Hz fourth order Butterworth low pass-filter to create a linear envelope for further analysis.

**Figure 1:**
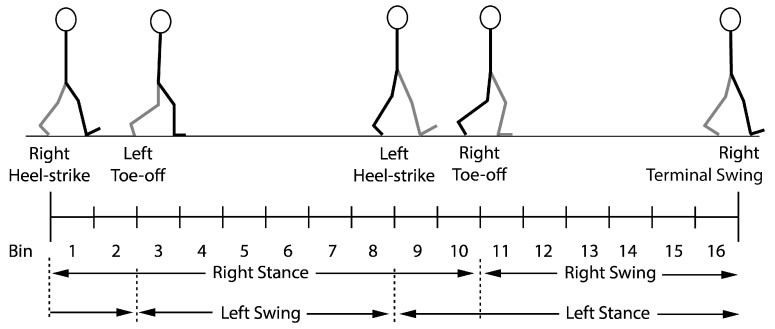
Adult gait cycle from right heel-strike to right termination of forward swing separated into sixteen equal phases.

EMG was sampled at 1000 Hz with a 16-bit A/D converter and collected and analyzed using custom-written LabView 2013 software (National Instruments Corp., Austin, TX, USA). EMG data for the walking trial were phase averaged into 16 equal phases across the gait cycle. Phases 1–10 correspond to stance, while phases 11–16 correspond to swing for the right leg (See Figure 1). EMG recording and processing methods follow the work of Hundza et al., 2018 [17].

### 2.4. Normalization of EMG Data

Averaged data for each phase were normalized using each of the three normalization techniques for each participant (see Section 2.5 for Formulae). Maximum EMG amplitude and mean EMG amplitude achieved across the phases during the walking task (EMG_Task_) were used for the EMG_Peak_ and EMG_Mean_ normalization techniques, respectively. The background (at rest) EMG was calculated from the mean EMG during a 500 ms window of the isometric submaximal contraction trial before the contraction began. The average load during the window of the isometric contraction was then divided by the subtracted EMG to create a scaling factor in units of Nm. To calculate EMG_TS_, each subject’s EMG_Task_ was multiplied by the scaling factor for that participant.

### 2.5. Formulae


$$\mathrm{Dynamic}\;\mathrm{Peak}\;\mathrm{Method}:{EMG}_{Peak}=\frac{{EMG}_{Task}}{{EMG}_{Task(Peak)}}$$

$$\mathrm{Dynamic}\;\mathrm{Mean}\;\mathrm{Method}:{EMG}_{Mean}=\frac{{EMG}_{Task}}{{EMG}_{Task(Mean)}}$$

$$\mathrm{Torque}\text{-}\mathrm{scaled}\;\mathrm{Method}:\;{EMG}_{TS}={EMG}_{Task}\frac{Torque\left(Nm\right)}{{EMG}_{Load}}$$

### 2.6. Statistical Analysis

Phase averaged data for EMG_Peak_, EMG_Mean_ and EMG_TS_ normalization techniques were compared. Separate repeated measures analysis of variance (ANOVA) tests for each of the 5 muscles and 3 normalization methods were conducted using a 2 (Age) × 16 (Phase) model with Tukey’s HSD post-hoc analysis. The coefficient of variation (CV) was calculated for all normalization methods for each cohort as a measure of inter-individual variability across phases. CV was compared between ages, normalization methods, and age*normalization method interactions for each muscle using repeated measures ANOVA with Tukey’s HSD post-hoc analysis. Pearson’s product–moment correlations were performed between cohorts across all phases. Significance was set at α < 0.05 for all comparisons and correlation coefficients were interpreted as <0.40 = weak, 0.40–0.69 = moderate, 0.70–0.89 = strong, and >0.90 very strong [18]. ANOVA effect sizes were reported as partial eta squared (np^2^) with values of 0.02 considered small, 0.13 as medium, and 0.26 as large [19]. All data are presented as mean ± SEM except subjects’ ages, which are presented as mean ± SD.

## 3. Results

### 3.1. Amplitude Differences

**Figure 2:**
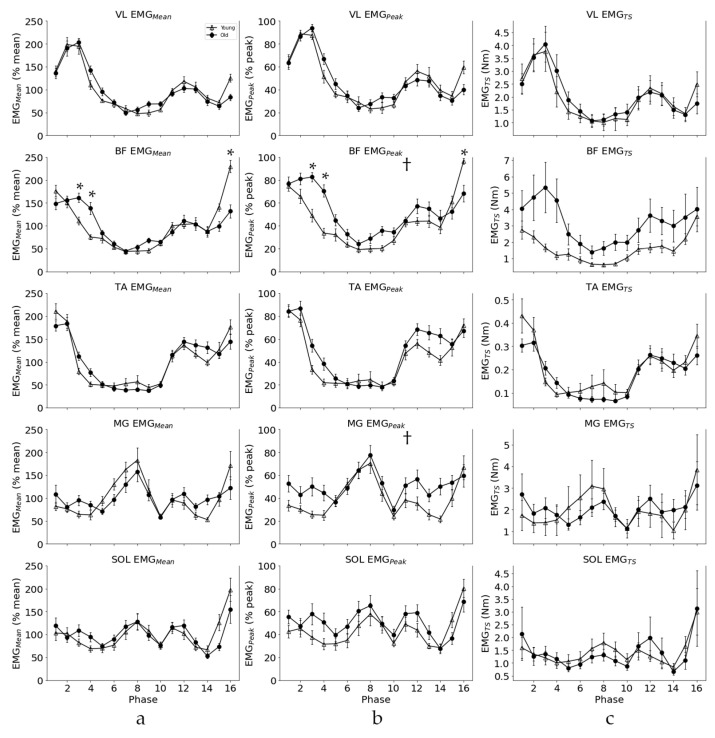
Normalized EMG averaged across YOUNG (N = 12) and OLD (N = 12) participants (+/− SEM) for EMG_Peak_ (**a**), EMG_Mean_ (**b**) and EMG_TS_ (**c**) for lower-limb muscles across the gait cycle during walking. Significant differences between cohorts at a given phase of movement are indicated by *, while † indicates a main effect for age.

Normalized EMG activity values averaged across participants across the phases of the gait cycle for YOUNG and OLD cohorts for each normalization method are displayed in Figure 2. The main effects for age were observed with a medium effect size in BF (F(1,22) = 6.266; *p* = 0.020, np^2^ = 0.21) and MG (F(1,22) = 6.631; *p* = 0.017, np^2^ = 0.14) for EMG_Peak_ normalization with a greater EMG amplitude for OLD (BF = 47.96 +/− 7.02; MG = 51.36 +/− 8.21) than YOUNG (BF = 43.93 +/− 6.88; MG = 48.62 +/− 8.66) cohorts. Age*phase interactions were observed with EMG_Mean_ and EMG_Peak_ in VL, BF, and TA and for EMG_TS_ in BF and TA with small effects observed for all comparisons except EMG_Mean_ and EMG_Peak_ in BF, which had a medium effect size (see Table 1).

Post-hoc Tukey’s test for age*phase interactions revealed that there were significant differences in the BF muscle with EMG_Mean_ normalization in phases 3 (*p* < 0.01), 4 (*p* < 0.001), and 16 (*p* < 0.05). There were also differences in the BF muscle with EMG_Peak_ normalization in phases 3 (*p* < 0.05), 4 (*p* < 0.01), and 16 (*p* < 0.001). With both normalization methods, the EMG amplitude was greater in OLD than YOUNG cohorts for phases 3 and 4 and greater in YOUNG than OLD cohorts for phase 16. Post-hoc analysis did not reveal differences between YOUNG and OLD cohorts for any other comparisons with interaction effects. The EMG amplitude for each age group can be seen for the five muscles across the phases of gait, including significant main and interaction effects, in Figure 2.

### 3.2. EMG Pattern

Normalized EMG activities for each cohort were compared for each muscle across the 16 phases of the gait cycle for each normalization method. There were very strong correlations observed for all comparisons between EMG_Mean_ and EMG_Peak_ for both cohorts with an average correlation across all muscles of r = 0.99 for OLD and r = 1.00 for YOUNG cohorts. EMG_Peak_ vs. EMG_TS_ were very strongly correlated for all muscles in the YOUNG cohort and for VL, BF, and TA in the OLD cohort with MG and SOL strongly correlated (average r = 0.88 for OLD and r = 0.98 for YOUNG). EMG_Mean_ vs. EMG_TS_ were very strongly correlated for all muscles in the YOUNG cohort and for VL, BF, and TA, and SOL in the OLD cohort with MG strongly correlated (average r = 0.90 for OLD and r = 0.98 for YOUNG). See Table 2 for all correlation results.

### 3.3. Inter-Subject Variability

**Figure 3:**
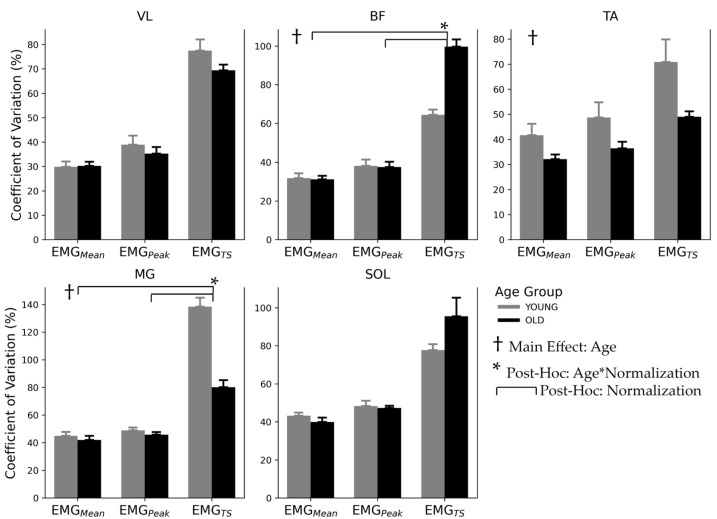
Mean coefficient of variation of three EMG normalization techniques for YOUNG (N = 12) and OLD (N = 12) participants in lower-limb muscles during walking. Significant main effect for age is indicated by †, significant age*normalization interactions by *, and significant post-hoc comparisons of normalization are indicated by horizontal bars.

The coefficient of variation (CV) across phases of the gait cycle averaged for each cohort and normalization technique for each muscle is displayed in Figure 3. The main effects for age were observed in BF, TA, and MG with a moderate effect size for BF, small effect size for TA, and large effect size for MG. The main effects with large effect sizes were observed for all normalization methods except TA, which had a medium effect size. There were age*normalization interactions with large effect sizes in BF and MG, while SOL had an age*normalization interaction with a small effect. See Table 3 for all significant results.

Post-hoc Tukey’s tests identified differences in CV between EMG_Peak_ vs. EMG_TS_ (*p* < 0.001) and EMG_Mean_ vs. EMG_TS_ (*p* < 0.001) with EMG_TS_ CV being higher in both cases, but with no difference between EMGMean and EMGPeak. Post-hoc analysis of the interaction between age and normalization effect between matched cases found that in BF, EMG_TS_ CV was greater in OLD than YOUNG (*p* < 0.001) cohorts and in MG, EMG_TS_ CV was greater in YOUNG than OLD (*p* < 0.001) cohorts.

## 4. Discussion

There were three main findings of this study comparing bioelectric (EMG_Mean_ and EMG_Peak_) and biomechanical (EMG_TS_) normalization techniques in older (OLD) and young (YOUNG) adults. First, there was no agreement for the statistical interpretation of EMG amplitude differences between age groups with the three normalization techniques. The EMG_Peak_ method identified amplitude differences between the two age groups in two of the five muscles (BF and MG) that EMG_Mean_ and EMG_TS_ did not. Additionally, both EMG_Peak_ and EMG_Mean_ normalization resulted in age*phase interactions in the biceps femoris muscle. This highlights the fact that the interpretation of EMG is dependent on the normalization method used, and it is essential to consider the strengths and limitations of each method when making functional interpretations.

The second notable finding was that EMG activation patterns across the gait cycle were generally similar between the normalization methods. As mentioned, the normalization method did result in notable EMG amplitude differences between cohorts for some muscles at some phases of the gait cycle, but the activation pattern across the phases were generally not different and they were strongly correlated for both cohorts.

Finally, the coefficient of variation (CV), the representation of inter-individual variability, was much greater for EMG_TS,_ while EMG_Mean_ and EMG_Peak_ were alike for all muscles. This finding supports our hypothesis that EMG_TS_ normalization would retain more inter-individual variability, which in some cases may be highly desirable; however, it also emphasizes that even when large differences are expected, a larger sample size may be required to counteract the increase in variability.

Our findings clearly demonstrate that the normalization method can influence the results and thus the conclusions drawn from EMG interpretation and highlights the importance of choosing a normalization technique based on the specific research question with a full understanding of the limitations and bias of the normalization procedure.

### 4.1. Rationale for Normalization Protocols

The bioelectric techniques, EMG_Mean_ and EMG_Peak_, were chosen for the present study as they are currently popular techniques [5]. Normalization to MVIC was excluded as a technique as it poses an injury, pain, or fear risk to an older cohort [20]. The biomechanical normalization method was designed to be easily performed across disparate populations (i.e., OLD and YOUNG cohorts) and include submaximal isometric contraction with absolute target torques at reproducible joint angles. Because the joint angle can influence force production and different populations may have different available ranges of motion, mid-range joint angles were chosen within an optimal force-joint angle range. Thus, this normalization method could be employed in any paradigm that was comparing EMG levels across disparate populations in different tasks. Isometric MVC is known to have equal inter-subject variability compared to dynamic submaximal contractions [6] and greater variability compared to isometric submaximal contractions [21]. Also, Burden et al. (2003) [6] reported that the use of dynamic (isokinetic) contraction methods does not decrease the intra-individual variability over isometric methods, while it does comparatively increase the complexity and duration of the normalization protocol. Thus, it is reasonable to infer that isometric submaximal contraction should have the lowest inter-subject variability. Finally, an absolute submaximal load for each muscle contraction was chosen to enable functionally relevant comparisons between disparate populations at the same absolute load [5,14].

While EMG_TS_ provides an individual scaling factor to normalize across subjects and populations, it does not account for the potential curvilinear relationship between muscle force and EMG during static contraction [22,23] and more complex relationships during dynamic contractions [24,25]. Importantly, the individual load scaling method presented in this study is a means to bring all subject-specific values to an approximate absolute load and is not meant to infer accurate measures of joint torques through EMG measurement. Therefore, as with all normalization techniques, functional interpretations of the results must account for these limitations.

### 4.2. EMG Activation Pattern and Amplitude

When comparing muscle activation across different populations, or before and after an intervention, it is valuable to evaluate both the amplitude and pattern of EMG activation as well as their interaction and consider the functional interpretation of the results. The differences observed between the OLD and YOUNG EMG amplitude across the phases of the gait cycle in the present study depend on the normalization technique used. Both EMG_Peak_ and EMG_Mean_ identified age*phase interactions in three muscles (VL, BF, and TA), while EMG_TS_ showed interactions in BF and TA, although following post-hoc analysis, significant age*phase interactions were only observed in BF for phases 3,4, and 16 with EMG_Peak_ and EMG_Mean_ normalization. These phases are critical transition points of the gait cycle in the BF muscle during weight acceptance in the stance phase (phases 3 and 4) and terminal swing (phase 16). In the stance phase, the pattern of EMG_TS_ generally agrees with both EMG_Mean_ and EMG_Peak_; however, terminal swing displays large differences where OLD EMG amplitude was greater than YOUNG using EMG_Peak_ and EMG_Mean_ normalization, while EMG_TS_ showed no evidence of differences (see Figure 2). This discrepancy leads to the potential for incorrect functional interpretations of results. For instance, one could infer that older adults have insufficient activation of BF during terminal swing based on the results of EMG_Peak_ and EMG_Mean_ and over-activation during the early stance phase. This phenomena is more likely a result of the normalization technique rather than a true functional difference between groups [5]. Thus, the normalization technique can influence the conclusions that can be drawn, which may have critical implications for studies attempting to support clinical differences between groups.

Despite the potential differences in EMG amplitude with the three normalization techniques, the muscle activation patterns across the gait cycle were generally similar between the cohorts across the normalization methods as evidenced by the significant correlations between OLD and YOUNG for each muscle. This indicates that each method effectively characterises the inherent phase modulation during gait.

### 4.3. Inter-Subject Variability

While EMG_Peak_ remains a common normalization technique, EMG_Mean_ has been suggested as the preferred bioelectric normalization method because it has been reported to reduce inter-individual variability more than EMG_Peak_ while maintaining the characteristic activation profile [6,7,26]. In a review by Burden (2010) [5], it was identified that EMG normalization procedures that reduce inter-subject variability are viewed positively because they increase the power of statistical comparisons between groups. However, normalization techniques that allow the expression of variability may more accurately represent the “true” variability in the EMG amplitude related to absolute force production during a task by subjects within a group. Therefore, it is important to consider the effect of a normalization technique on the inter-subject variability inherent in the raw EMG as well as maintaining variability associated with “true” differences in EMG amplitude relative to absolute force production during task performance. This is aptly demonstrated when comparing the effect of normalization using EMG_TS_ compared to the EMG_Mean_ and EMG_Peak_. This can be observed in the larger CV values for EMG_TS_ compared to EMG_Mean_ and EMG_Peak_ for all muscles (see Figure 3). The EMG_TS_ method takes into account the subjects’ capacity relative to an absolute load when comparing EMG during the walking task, whereas EMG_Mean_ and EMG_Peak_ take into account the subjects’ performance relative to a trial-specific EMG. However, greater variability with EMG_TS_ results in reduced statistical power and likely warrants larger sample sizes than either of the bioelectrical normalization techniques.

A potential source of error that could increase inter-individual variability in torque-scaled normalization is the accuracy of the force generated by the participant. Even small deviations from the target force could result in large changes in the Nm/µV estimate. This phenomenon was also observed by Yang and Winter (1984) [7] who used 50% MVIC–moment relationships to normalize EMG and found that the accuracy of the submaximal contraction was important as small deviations from 50% resulted in large errors in their scaling factor, particularly if the EMG–moment relationship was not linear. Therefore, it remains to be confirmed if increased variability with load scale normalization truly represents actual differences among individuals or if methodological rigour contributes to this variability. The effects of age on variability of force production and motor unit discharge patterns imply that older individuals have intrinsically higher variability in force production [27]. This is supported by our finding that OLD CV was larger than YOUNG CV when normalized to EMG_TS_ for BF where OLD CV was greater than YOUNG CV, but our results observed the opposite in MG where YOUNG CV was greater than OLD CV. No age differences in CV were observed with EMG_Mean_ and EMG_Peak_ methods. Since the bioelectric normalization methods intentionally suppress inter-individual variability, this result is not unexpected [5]. This highlights the concerns raised by Hsu et al. (2006) [28] that normalizing EMG to the mean or peak of a task reduces inter-individual variability and dilutes the true variation in gait EMG and does not represent a subject’s actual capacity, but rather the EMG activation relative to the task-specific EMG value.

## 5. Conclusions

The current findings demonstrate that depending on the normalization technique employed, different results emerge from the same raw EMG data, leading to the potential for different conclusions to be drawn. Thus, it is critical to have an in depth understanding of the influence of the normalization method used to make an informed choice of the most appropriate method to address the research question and accurately interpret the results. We noted the differing effect of the normalization method on EMG amplitude and inter-subject variability. Additionally, although the activation patterns across phases were generally similar between normalization methods, there were notable differences between cohorts in some muscles depending on the normalization technique used.

## Figures and Tables

**Table 1:** Significant age*phase interactions between OLD (N = 12) and YOUNG (N = 12) mean EMG across the gait cycle (16 phases) in five muscles involved in walking.

Muscle	Normalization	df	*p*	F	np^2^
VL	EMG_Mean_	15, 330	0.036	1.781	0.08
VL	EMG_Peak_	15, 330	0.003	2.334	0.10
BF	EMG_Mean_	15, 330	0.000	7.333	0.25
BF	EMG_Peak_	15, 330	0.000	6.088	0.22
BF	EMG_TS_	15, 330	0.001	2.592	0.11
TA	EMG_Mean_	15, 330	0.046	1.721	0.07
TA	EMG_Peak_	15, 330	0.007	2.170	0.09
TA	EMG_TS_	15, 330	0.032	1.814	0.08

**Table 2:** Pearson’s correlations between OLD (N = 12) and YOUNG (N = 12) mean EMG across the gait cycle (16 phases) in five muscles involved in walking.

Age	Correlation	VL	BF	TA	MG	SOL
OLD	EMG_Peak_ vs. EMG_Mean_	1.00	1.00	1.00	0.99	0.98
OLD	EMG_Peak_ vs. EMG_TS_	0.99	0.98	1.00	0.70	0.72
OLD	EMG_Mean_ vs. EMG_TS_	0.99	0.98	1.00	0.70	0.84
YOUNG	EMG_Peak_ vs. EMG_Mean_	1.00	1.00	1.00	1.00	0.99
YOUNG	EMG_Peak_ vs. EMG_TS_	1.00	1.00	0.99	0.93	0.97
YOUNG	EMG_Mean_ vs. EMG_TS_	1.00	1.00	0.99	0.92	0.98

**Table 3:** Significant age, normalization, and age*normalization interactions between OLD (N = 12) and YOUNG (N = 12) EMG coefficient of variation across the gait cycle (16 phases) in five muscles involved in walking.

Effect	Muscle	df	*p*	F	np2
Age	BF	1, 90	0.000	23.667	0.21
Age	TA	1, 90	0.001	12.470	0.12
Age	MG	1, 90	0.000	44.749	0.33
Normalization	VL	2, 90	0.000	119.460	0.73
Normalization	BF	2, 90	0.000	185.955	0.81
Normalization	TA	2, 90	0.000	11.348	0.20
Normalization	MG	2, 90	0.000	176.677	0.80
Normalization	SOL	2, 90	0.000	57.755	0.56
Age*Normalization	BF	2, 90	0.000	26.188	0.37
Age*Normalization	MG	2, 90	0.000	32.780	0.42
Age*Normalization	SOL	2, 90	0.043	0.821	0.02

## Data Availability

Data used in this study cannot be shared outside of the original research group.
